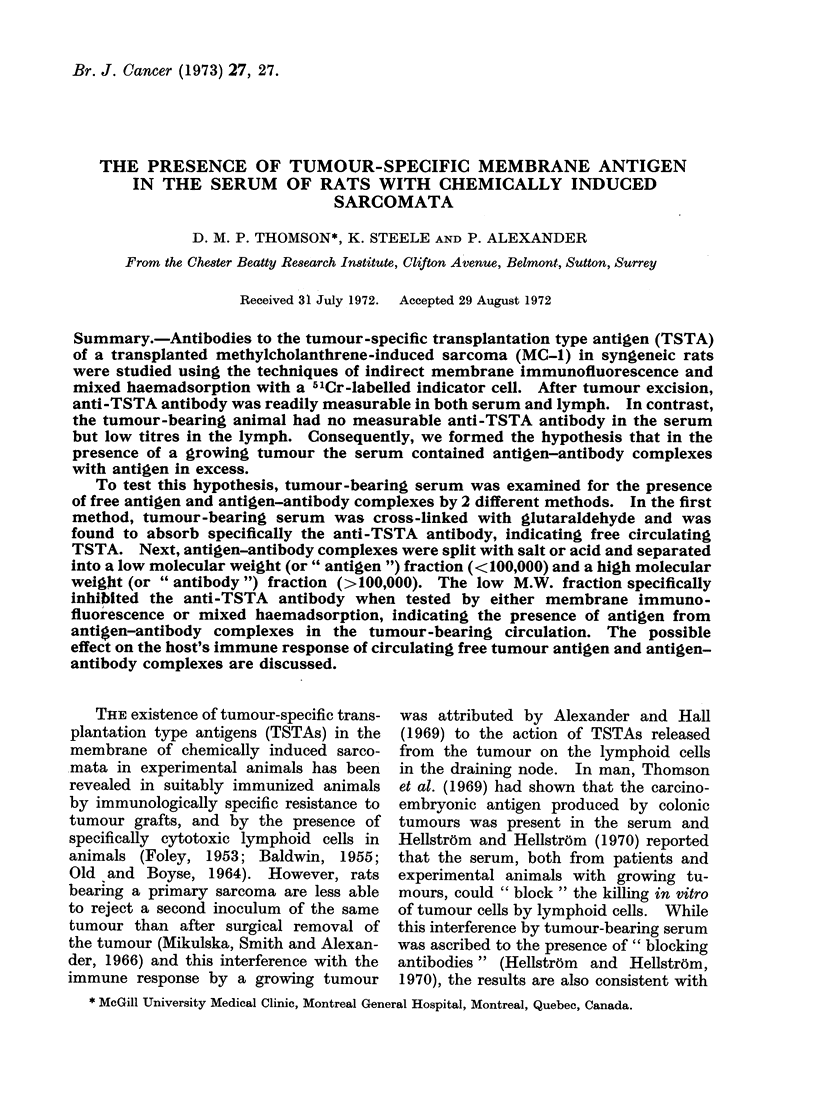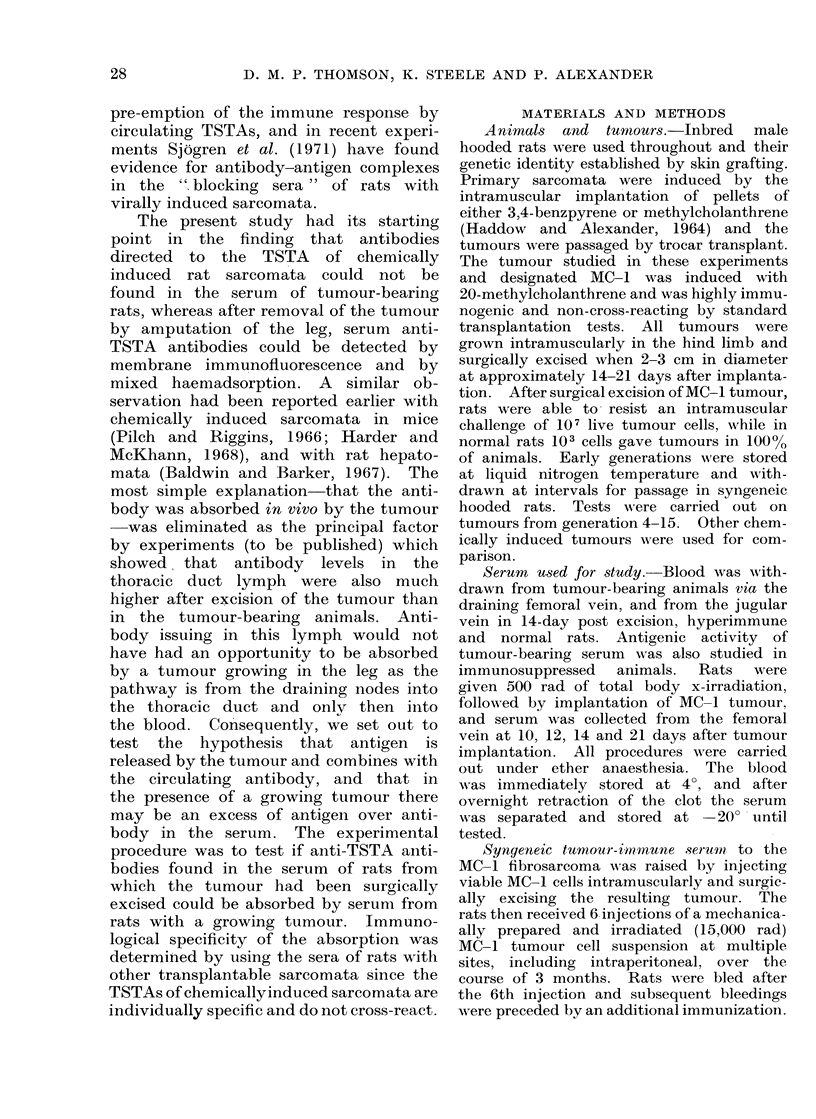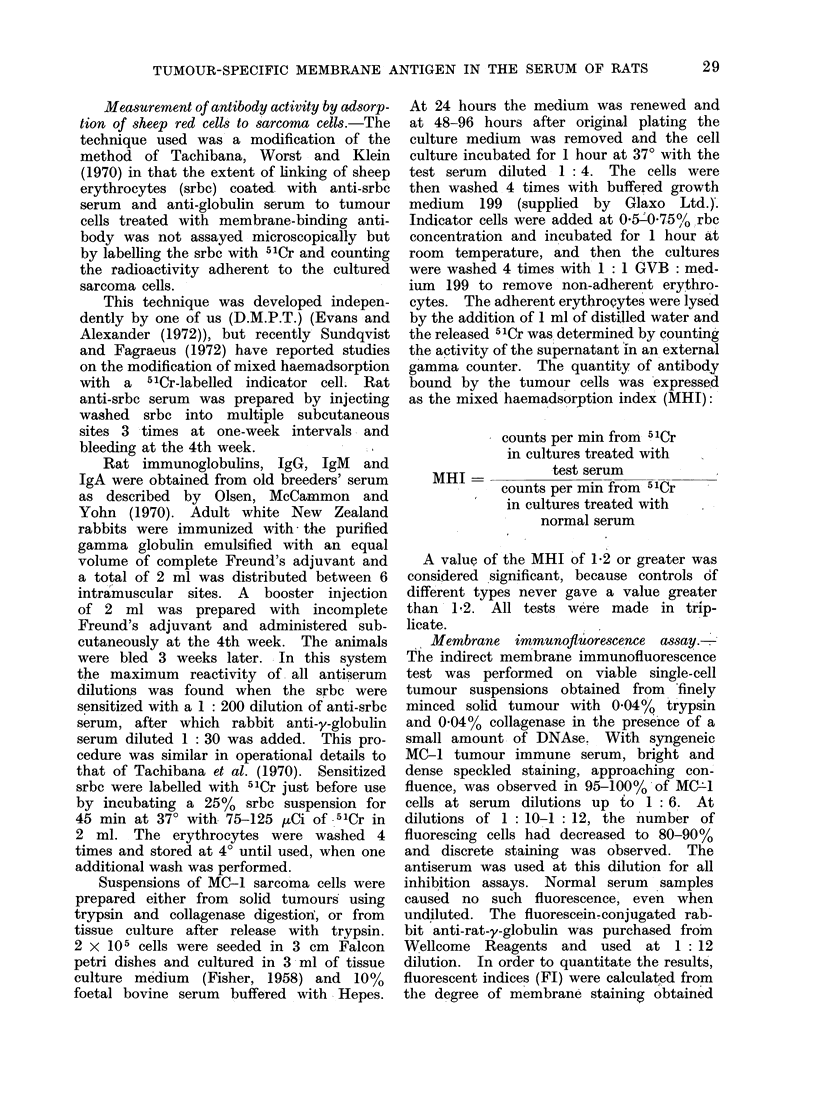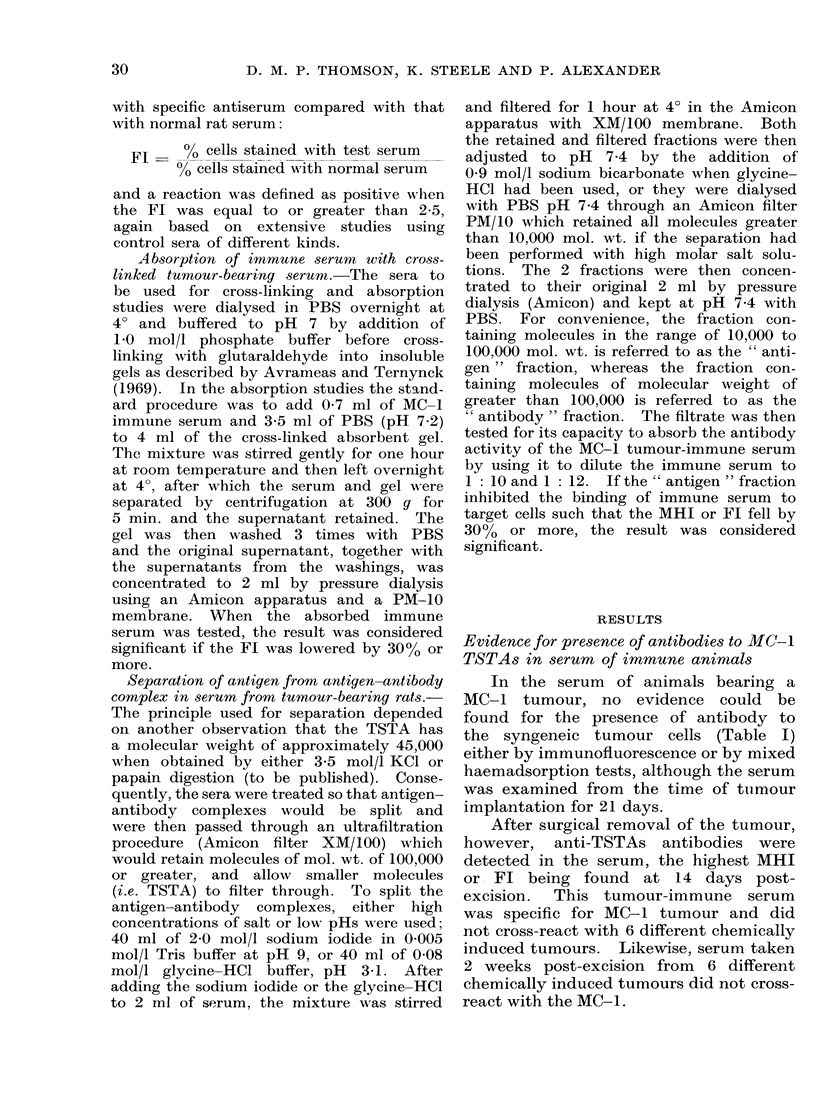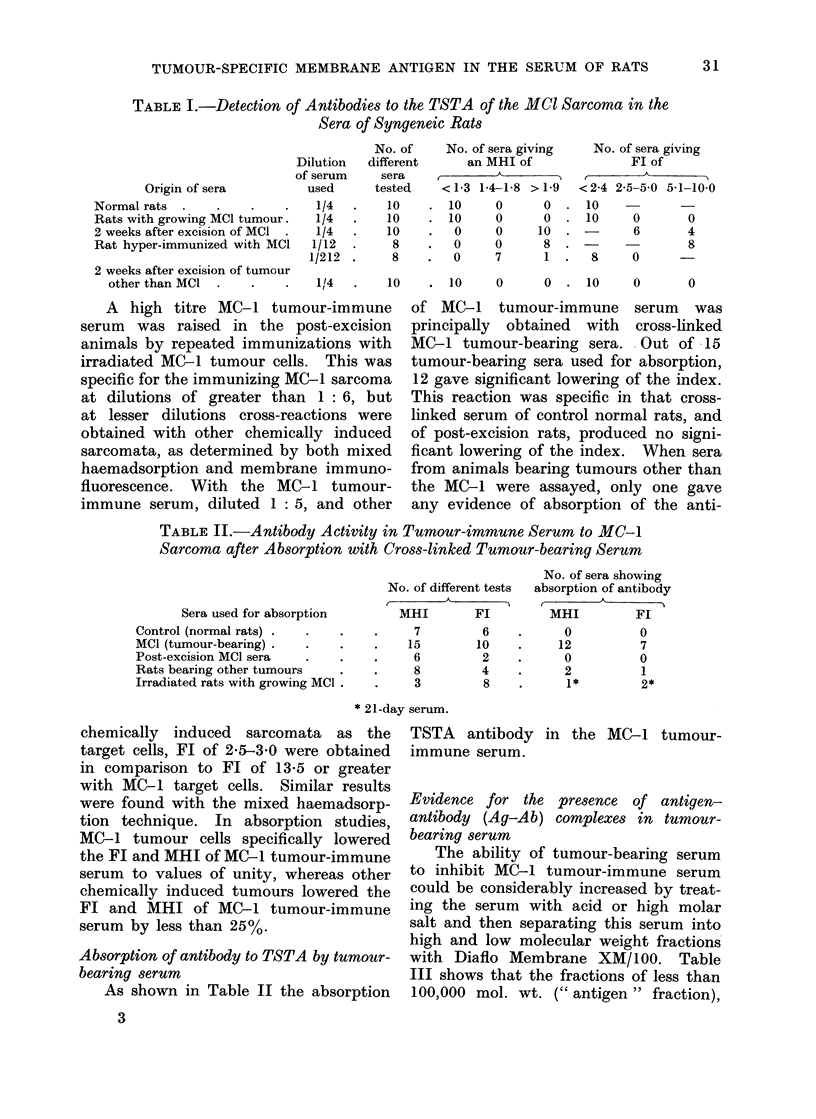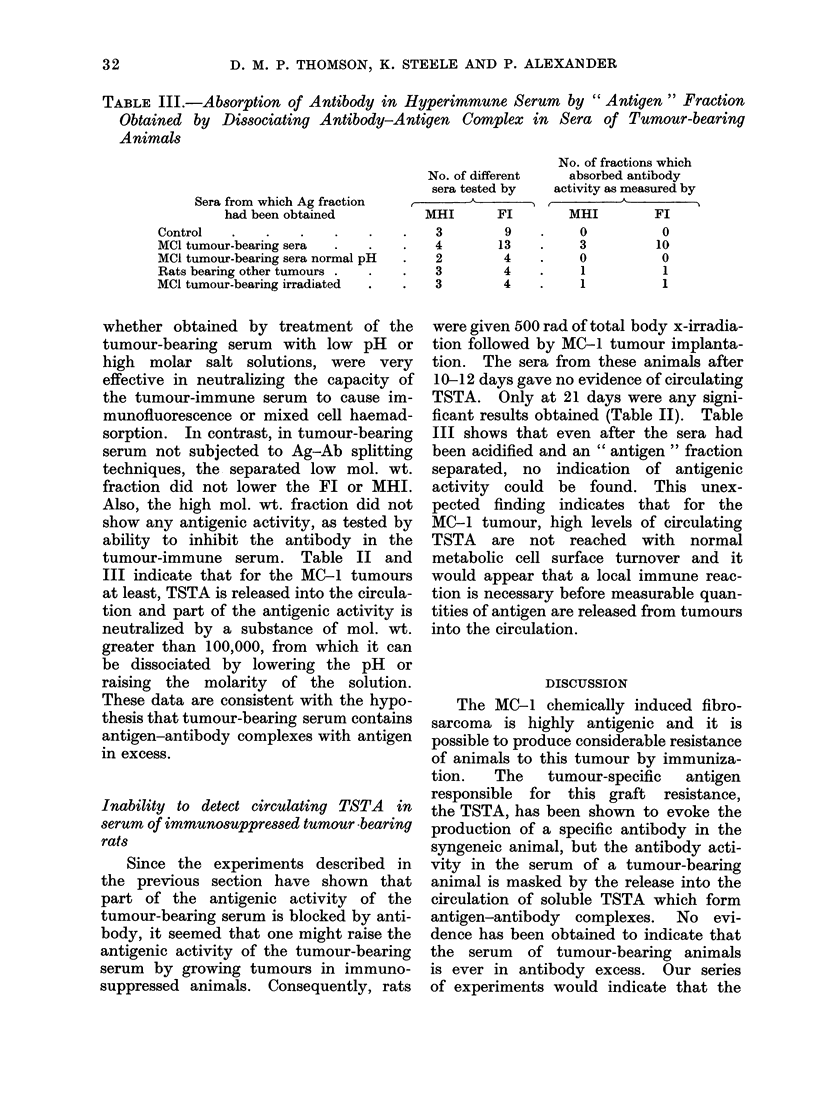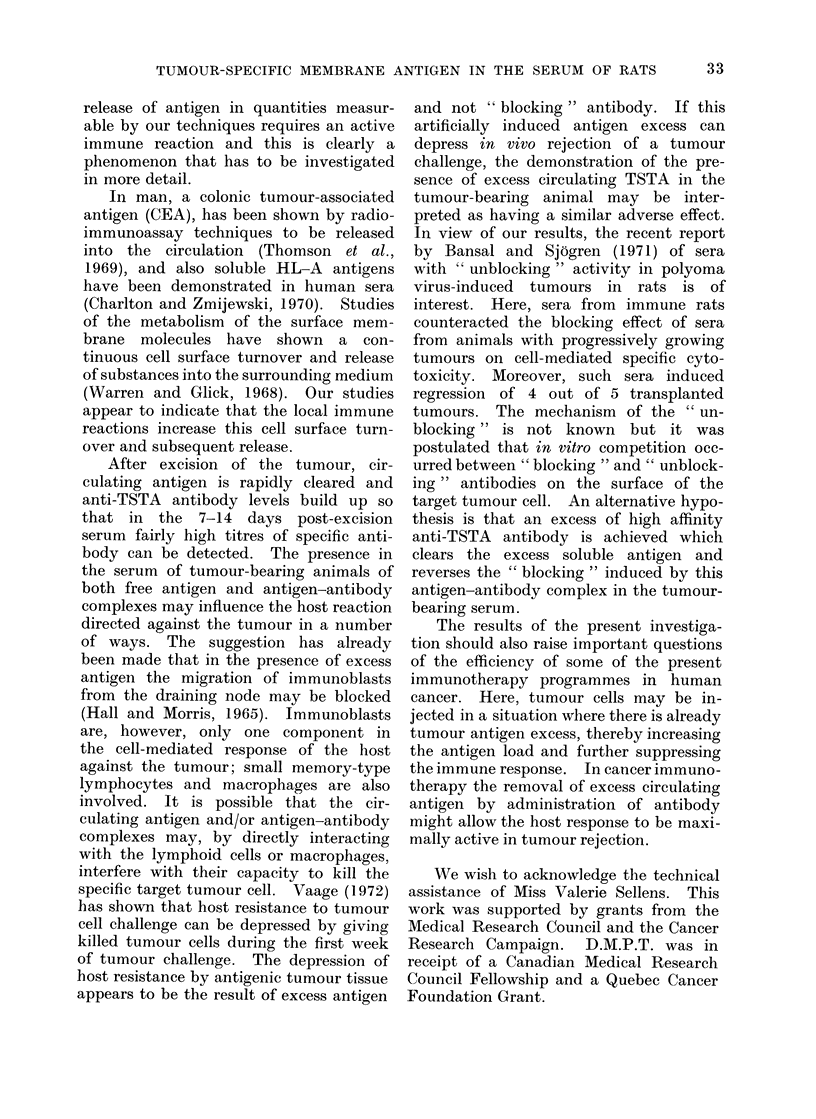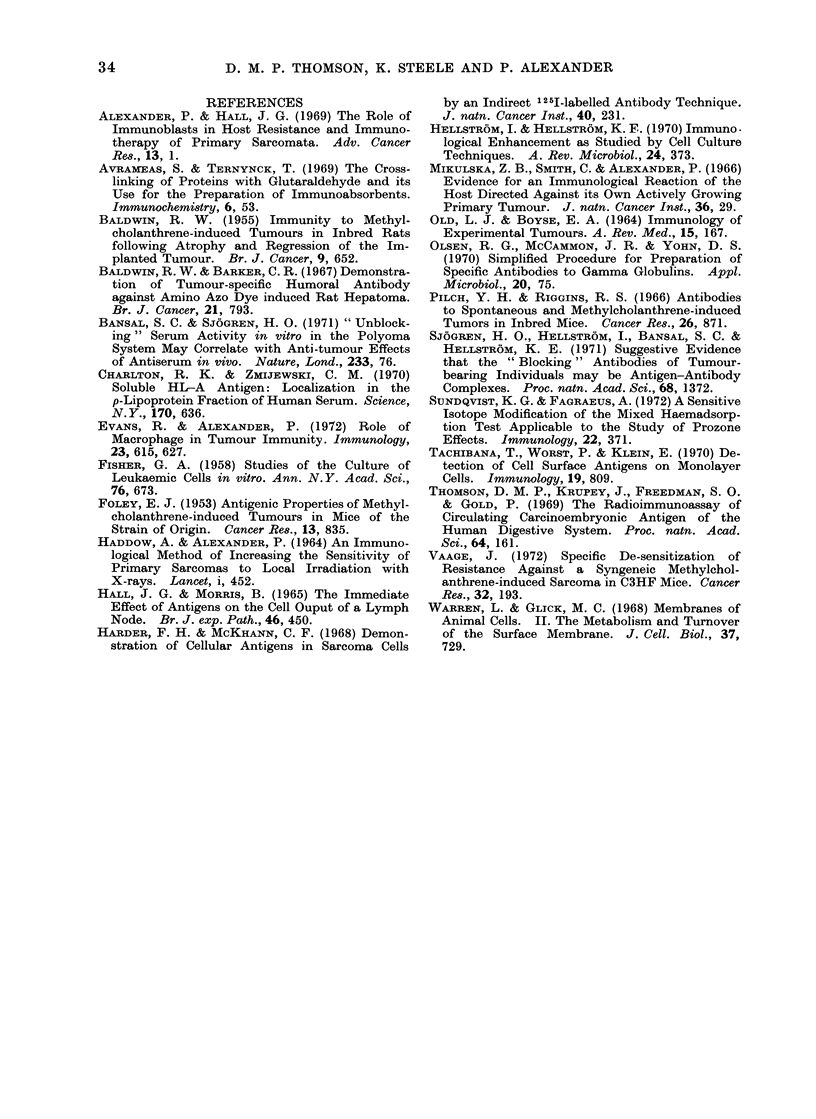# The Presence of Tumour Specific Membrane Antigen in the Serum of Rats with Chemically Induced Sarcomata

**DOI:** 10.1038/bjc.1973.4

**Published:** 1973-01

**Authors:** D. M. P. Thomson, K. Steele, P. Alexander

## Abstract

Antibodies to the tumour-specific transplantation type antigen (TSTA) of a transplanted methylcholanthrene-induced sarcoma (MC-1) in syngeneic rats were studied using the techniques of indirect membrane immunofluorescence and mixed haemadsorption with a ^51^Cr-labelled indicator cell. After tumour excision, anti-TSTA antibody was readily measurable in both serum and lymph. In contrast, the tumour-bearing animal had no measurable anti-TSTA antibody in the serum but low titres in the lymph. Consequently, we formed the hypothesis that in the presence of a growing tumour the serum contained antigen-antibody complexes with antigen in excess.

To test this hypothesis, tumour-bearing serum was examined for the presence of free antigen and antigen-antibody complexes by 2 different methods. In the first method, tumour-bearing serum was cross-linked with glutaraldehyde and was found to absorb specifically the anti-TSTA antibody, indicating free circulating TSTA. Next, antigen-antibody complexes were split with salt or acid and separated into a low molecular weight (or “antigen”) fraction (<100,000) and a high molecular weight (or “antibody”) fraction (>100,000). The low M.W. fraction specifically inhibited the anti-TSTA antibody when tested by either membrane immunofluorescence or mixed haemadsorption, indicating the presence of antigen from antigen-antibody complexes in the tumour-bearing circulation. The possible effect on the host's immune response of circulating free tumour antigen and antigen-antibody complexes are discussed.


*Br. J. Cancer* (1973) **27**, 27.

### THE PRESENCE OF TUMOUR-SPECIFIC MEMBRANE ANTIGEN IN THE SERUM OF RATS WITH CHEMICALLY INDUCED SARCOMATA

D. M. P. THOMSON*, K. STEELE AND P. ALEXANDER

*From the Chester Beatty Research Institute, Clifton Avenue, Belmont, Sutton, Surrey*

Received 31 July 1972. Accepted 29 August 1972

**Summary.—Antibodies to the tumour-specific transplantation type antigen (TSTA) of a transplanted methylcholanthrene-induced sarcoma (MC–1) in syngeneic rats were studied using the techniques of indirect membrane immunofluorescence and mixed haemadsorption with a $^{51}$Cr-labelled indicator cell. After tumour excision, anti-TSTA antibody was readily measurable in both serum and lymph. In contrast, the tumour-bearing animal had no measurable anti-TSTA antibody in the serum but low titres in the lymph. Consequently, we formed the hypothesis that in the presence of a growing tumour the serum contained antigen–antibody complexes with antigen in excess.**

**To test this hypothesis, tumour-bearing serum was examined for the presence of free antigen and antigen–antibody complexes by 2 different methods. In the first method, tumour-bearing serum was cross-linked with glutaraldehyde and was found to absorb specifically the anti-TSTA antibody, indicating free circulating TSTA. Next, antigen–antibody complexes were split with salt or acid and separated into a low molecular weight (or " antigen ") fraction (<100,000) and a high molecular weight (or " antibody ") fraction (>100,000). The low M.W. fraction specifically inhibited the anti-TSTA antibody when tested by either membrane immunofluorescence or mixed haemadsorption, indicating the presence of antigen from antigen–antibody complexes in the tumour-bearing circulation. The possible effect on the host's immune response of circulating free tumour antigen and antigen–antibody complexes are discussed.**

THE existence of tumour-specific transplantation type antigens (TSTAs) in the membrane of chemically induced sarcomata in experimental animals has been revealed in suitably immunized animals by immunologically specific resistance to tumour grafts, and by the presence of specifically cytotoxic lymphoid cells in animals (Foley, 1953; Baldwin, 1955; Old and Boyse, 1964). However, rats bearing a primary sarcoma are less able to reject a second inoculum of the same tumour than after surgical removal of the tumour (Mikulska, Smith and Alexander, 1966) and this interference with the immune response by a growing tumour was attributed by Alexander and Hall (1969) to the action of TSTAs released from the tumour on the lymphoid cells in the draining node. In man, Thomson *et al.* (1969) had shown that the carcino-embryonic antigen produced by colonic tumours was present in the serum and Hellström and Hellström (1970) reported that the serum, both from patients and experimental animals with growing tumours, could " block " the killing *in vitro* of tumour cells by lymphoid cells. While this interference by tumour-bearing serum was ascribed to the presence of " blocking antibodies " (Hellström and Hellström, 1970), the results are also consistent with

* McGill University Medical Clinic, Montreal General Hospital, Montreal, Quebec, Canada.

pre-emption of the immune response by circulating TSTAs, and in recent experiments Sjögren *et al.* (1971) have found evidence for antibody–antigen complexes in the "blocking sera" of rats with virally induced sarcomata.

The present study had its starting point in the finding that antibodies directed to the TSTA of chemically induced rat sarcomata could not be found in the serum of tumour-bearing rats, whereas after removal of the tumour by amputation of the leg, serum anti-TSTA antibodies could be detected by membrane immunofluorescence and by mixed haemadsorption. A similar observation had been reported earlier with chemically induced sarcomata in mice (Pilch and Riggins, 1966; Harder and McKhann, 1968), and with rat hepatomata (Baldwin and Barker, 1967). The most simple explanation—that the antibody was absorbed *in vivo* by the tumour—was eliminated as the principal factor by experiments (to be published) which showed that antibody levels in the thoracic duct lymph were also much higher after excision of the tumour than in the tumour-bearing animals. Antibody issuing in this lymph would not have had an opportunity to be absorbed by a tumour growing in the leg as the pathway is from the draining nodes into the thoracic duct and only then into the blood. Consequently, we set out to test the hypothesis that antigen is released by the tumour and combines with the circulating antibody, and that in the presence of a growing tumour there may be an excess of antigen over antibody in the serum. The experimental procedure was to test if anti-TSTA antibodies found in the serum of rats from which the tumour had been surgically excised could be absorbed by serum from rats with a growing tumour. Immunological specificity of the absorption was determined by using the sera of rats with other transplantable sarcomata since the TSTAs of chemically induced sarcomata are individually specific and do not cross-react.

### MATERIALS AND METHODS

*Animals and tumours.*—Inbred male hooded rats were used throughout and their genetic identity established by skin grafting. Primary sarcomata were induced by the intramuscular implantation of pellets of either 3,4-benzpyrene or methylcholanthrene (Haddow and Alexander, 1964) and the tumours were passaged by trocar transplant. The tumour studied in these experiments and designated MC–1 was induced with 20-methylcholanthrene and was highly immunogenic and non-cross-reacting by standard transplantation tests. All tumours were grown intramuscularly in the hind limb and surgically excised when 2–3 cm in diameter at approximately 14–21 days after implantation. After surgical excision of MC–1 tumour, rats were able to resist an intramuscular challenge of $10^7$ live tumour cells, while in normal rats $10^3$ cells gave tumours in 100% of animals. Early generations were stored at liquid nitrogen temperature and withdrawn at intervals for passage in syngeneic hooded rats. Tests were carried out on tumours from generation 4–15. Other chemically induced tumours were used for comparison.

*Serum used for study.*—Blood was withdrawn from tumour-bearing animals *via* the draining femoral vein, and from the jugular vein in 14-day post excision, hyperimmune and normal rats. Antigenic activity of tumour-bearing serum was also studied in immunosuppressed animals. Rats were given 500 rad of total body x-irradiation, followed by implantation of MC–1 tumour, and serum was collected from the femoral vein at 10, 12, 14 and 21 days after tumour implantation. All procedures were carried out under ether anaesthesia. The blood was immediately stored at 4°, and after overnight retraction of the clot the serum was separated and stored at −20° until tested.

*Syngeneic tumour-immune serum* to the MC–1 fibrosarcoma was raised by injecting viable MC–1 cells intramuscularly and surgically excising the resulting tumour. The rats then received 6 injections of a mechanically prepared and irradiated (15,000 rad) MC–1 tumour cell suspension at multiple sites, including intraperitoneal, over the course of 3 months. Rats were bled after the 6th injection and subsequent bleedings were preceded by an additional immunization.

*Measurement of antibody activity by adsorption of sheep red cells to sarcoma cells.*—The technique used was a modification of the method of Tachibana, Worst and Klein (1970) in that the extent of linking of sheep erythrocytes (srbc) coated with anti-srbc serum and anti-globulin serum to tumour cells treated with membrane-binding antibody was not assayed microscopically but by labelling the srbc with $^{51}Cr$ and counting the radioactivity adherent to the cultured sarcoma cells.

This technique was developed independently by one of us (D.M.P.T.) (Evans and Alexander (1972)), but recently Sundqvist and Fagraeus (1972) have reported studies on the modification of mixed haemadsorption with a $^{51}Cr$-labelled indicator cell. Rat anti-srbc serum was prepared by injecting washed srbc into multiple subcutaneous sites 3 times at one-week intervals and bleeding at the 4th week.

Rat immunoglobulins, IgG, IgM and IgA were obtained from old breeders' serum as described by Olsen, McCammon and Yohn (1970). Adult white New Zealand rabbits were immunized with the purified gamma globulin emulsified with an equal volume of complete Freund's adjuvant and a total of 2 ml was distributed between 6 intramuscular sites. A booster injection of 2 ml was prepared with incomplete Freund's adjuvant and administered subcutaneously at the 4th week. The animals were bled 3 weeks later. In this system the maximum reactivity of all antiserum dilutions was found when the srbc were sensitized with a 1 : 200 dilution of anti-srbc serum, after which rabbit anti-$\gamma$-globulin serum diluted 1 : 30 was added. This procedure was similar in operational details to that of Tachibana *et al.* (1970). Sensitized srbc were labelled with $^{51}Cr$ just before use by incubating a 25% srbc suspension for 45 min at 37° with 75–125 $\mu$Ci of $^{51}Cr$ in 2 ml. The erythrocytes were washed 4 times and stored at 4° until used, when one additional wash was performed.

Suspensions of MC–1 sarcoma cells were prepared either from solid tumours using trypsin and collagenase digestion, or from tissue culture after release with trypsin. $2 \times 10^5$ cells were seeded in 3 cm Falcon petri dishes and cultured in 3 ml of tissue culture medium (Fisher, 1958) and 10% foetal bovine serum buffered with Hepes. At 24 hours the medium was renewed and at 48–96 hours after original plating the culture medium was removed and the cell culture incubated for 1 hour at 37° with the test serum diluted 1 : 4. The cells were then washed 4 times with buffered growth medium 199 (supplied by Glaxo Ltd.). Indicator cells were added at 0·5–0·75% rbc concentration and incubated for 1 hour at room temperature, and then the cultures were washed 4 times with 1 : 1 GVB : medium 199 to remove non-adherent erythrocytes. The adherent erythrocytes were lysed by the addition of 1 ml of distilled water and the released $^{51}Cr$ was determined by counting the activity of the supernatant in an external gamma counter. The quantity of antibody bound by the tumour cells was expressed as the mixed haemadsorption index (MHI):

$$\text{MHI} = \frac{\begin{array}{c}\text{counts per min from } ^{51}\text{Cr} \\ \text{in cultures treated with} \\ \text{test serum}\end{array}}{\begin{array}{c}\text{counts per min from } ^{51}\text{Cr} \\ \text{in cultures treated with} \\ \text{normal serum}\end{array}}$$

A value of the MHI of 1·2 or greater was considered significant, because controls of different types never gave a value greater than 1·2. All tests were made in triplicate.

*Membrane immunofluorescence assay.*—The indirect membrane immunofluorescence test was performed on viable single-cell tumour suspensions obtained from finely minced solid tumour with 0·04% trypsin and 0·04% collagenase in the presence of a small amount of DNAse. With syngeneic MC–1 tumour immune serum, bright and dense speckled staining, approaching confluence, was observed in 95–100% of MC–1 cells at serum dilutions up to 1 : 6. At dilutions of 1 : 10–1 : 12, the number of fluorescing cells had decreased to 80–90% and discrete staining was observed. The antiserum was used at this dilution for all inhibition assays. Normal serum samples caused no such fluorescence, even when undiluted. The fluorescein-conjugated rabbit anti-rat-$\gamma$-globulin was purchased from Wellcome Reagents and used at 1 : 12 dilution. In order to quantitate the results, fluorescent indices (FI) were calculated from the degree of membrane staining obtained with specific antiserum compared with that with normal rat serum:

$$FI = \frac{\% \text{ cells stained with test serum}}{\% \text{ cells stained with normal serum}}$$

and a reaction was defined as positive when the FI was equal to or greater than 2·5, again based on extensive studies using control sera of different kinds.

*Absorption of immune serum with cross-linked tumour-bearing serum.*—The sera to be used for cross-linking and absorption studies were dialysed in PBS overnight at 4° and buffered to pH 7 by addition of 1·0 mol/l phosphate buffer before cross-linking with glutaraldehyde into insoluble gels as described by Avrameas and Ternynck (1969). In the absorption studies the standard procedure was to add 0·7 ml of MC–1 immune serum and 3·5 ml of PBS (pH 7·2) to 4 ml of the cross-linked absorbent gel. The mixture was stirred gently for one hour at room temperature and then left overnight at 4°, after which the serum and gel were separated by centrifugation at 300 *g* for 5 min. and the supernatant retained. The gel was then washed 3 times with PBS and the original supernatant, together with the supernatants from the washings, was concentrated to 2 ml by pressure dialysis using an Amicon apparatus and a PM–10 membrane. When the absorbed immune serum was tested, the result was considered significant if the FI was lowered by 30% or more.

*Separation of antigen from antigen–antibody complex in serum from tumour-bearing rats.*—The principle used for separation depended on another observation that the TSTA has a molecular weight of approximately 45,000 when obtained by either 3·5 mol/l KCl or papain digestion (to be published). Consequently, the sera were treated so that antigen–antibody complexes would be split and were then passed through an ultrafiltration procedure (Amicon filter XM/100) which would retain molecules of mol. wt. of 100,000 or greater, and allow smaller molecules (*i.e.* TSTA) to filter through. To split the antigen–antibody complexes, either high concentrations of salt or low pHs were used; 40 ml of 2·0 mol/l sodium iodide in 0·005 mol/l Tris buffer at pH 9, or 40 ml of 0·08 mol/l glycine–HCl buffer, pH 3·1. After adding the sodium iodide or the glycine–HCl to 2 ml of serum, the mixture was stirred and filtered for 1 hour at 4° in the Amicon apparatus with XM/100 membrane. Both the retained and filtered fractions were then adjusted to pH 7·4 by the addition of 0·9 mol/l sodium bicarbonate when glycine–HCl had been used, or they were dialysed with PBS pH 7·4 through an Amicon filter PM/10 which retained all molecules greater than 10,000 mol. wt. if the separation had been performed with high molar salt solutions. The 2 fractions were then concentrated to their original 2 ml by pressure dialysis (Amicon) and kept at pH 7·4 with PBS. For convenience, the fraction containing molecules in the range of 10,000 to 100,000 mol. wt. is referred to as the "antigen" fraction, whereas the fraction containing molecules of molecular weight of greater than 100,000 is referred to as the "antibody" fraction. The filtrate was then tested for its capacity to absorb the antibody activity of the MC–1 tumour-immune serum by using it to dilute the immune serum to 1 : 10 and 1 : 12. If the "antigen" fraction inhibited the binding of immune serum to target cells such that the MHI or FI fell by 30% or more, the result was considered significant.

### RESULTS

### *Evidence for presence of antibodies to MC–1 TSTAs in serum of immune animals*

In the serum of animals bearing a MC–1 tumour, no evidence could be found for the presence of antibody to the syngeneic tumour cells (Table I) either by immunofluorescence or by mixed haemadsorption tests, although the serum was examined from the time of tumour implantation for 21 days.

After surgical removal of the tumour, however, anti-TSTAs antibodies were detected in the serum, the highest MHI or FI being found at 14 days post-excision. This tumour-immune serum was specific for MC–1 tumour and did not cross-react with 6 different chemically induced tumours. Likewise, serum taken 2 weeks post-excision from 6 different chemically induced tumours did not cross-react with the MC–1.

TABLE I.—*Detection of Antibodies to the TSTA of the MCl Sarcoma in the Sera of Syngeneic Rats*

| Origin of sera | Dilution of serum used | No. of different sera tested | No. of sera giving an MHI of | | | No. of sera giving FI of | | |
|---|---|---|---|---|---|---|---|---|
| | | | < 1·3 | 1·4–1·8 | > 1·9 | < 2·4 | 2·5–5·0 | 5·1–10·0 |
| Normal rats | 1/4 | 10 | 10 | 0 | 0 | 10 | — | — |
| Rats with growing MCl tumour | 1/4 | 10 | 10 | 0 | 0 | 10 | 0 | 0 |
| 2 weeks after excision of MCl | 1/4 | 10 | 0 | 0 | 10 | — | 6 | 4 |
| Rat hyper-immunized with MCl | 1/12 | 8 | 0 | 0 | 8 | — | — | 8 |
| | 1/212 | 8 | 0 | 7 | 1 | 8 | 0 | — |
| 2 weeks after excision of tumour other than MCl | 1/4 | 10 | 10 | 0 | 0 | 10 | 0 | 0 |

A high titre MC–1 tumour-immune serum was raised in the post-excision animals by repeated immunizations with irradiated MC–1 tumour cells. This was specific for the immunizing MC–1 sarcoma at dilutions of greater than 1 : 6, but at lesser dilutions cross-reactions were obtained with other chemically induced sarcomata, as determined by both mixed haemadsorption and membrane immunofluorescence. With the MC–1 tumour-immune serum, diluted 1 : 5, and other chemically induced sarcomata as the target cells, FI of 2·5–3·0 were obtained in comparison to FI of 13·5 or greater with MC–1 target cells. Similar results were found with the mixed haemadsorption technique. In absorption studies, MC–1 tumour cells specifically lowered the FI and MHI of MC–1 tumour-immune serum to values of unity, whereas other chemically induced tumours lowered the FI and MHI of MC–1 tumour-immune serum by less than 25%.

*Absorption of antibody to TSTA by tumour-bearing serum*

As shown in Table II the absorption of MC–1 tumour-immune serum was principally obtained with cross-linked MC–1 tumour-bearing sera. Out of 15 tumour-bearing sera used for absorption, 12 gave significant lowering of the index. This reaction was specific in that cross-linked serum of control normal rats, and of post-excision rats, produced no significant lowering of the index. When sera from animals bearing tumours other than the MC–1 were assayed, only one gave any evidence of absorption of the anti-TSTA antibody in the MC–1 tumour-immune serum.

TABLE II.—*Antibody Activity in Tumour-immune Serum to MC–1 Sarcoma after Absorption with Cross-linked Tumour-bearing Serum*

| Sera used for absorption | No. of different tests | | No. of sera showing absorption of antibody | |
|---|---|---|---|---|
| | MHI | FI | MHI | FI |
| Control (normal rats) | 7 | 6 | 0 | 0 |
| MCl (tumour-bearing) | 15 | 10 | 12 | 7 |
| Post-excision MCl sera | 6 | 2 | 0 | 0 |
| Rats bearing other tumours | 8 | 4 | 2 | 1 |
| Irradiated rats with growing MCl | 3 | 8 | 1* | 2* |

* 21-day serum.

*Evidence for the presence of antigen–antibody (Ag–Ab) complexes in tumour-bearing serum*

The ability of tumour-bearing serum to inhibit MC–1 tumour-immune serum could be considerably increased by treating the serum with acid or high molar salt and then separating this serum into high and low molecular weight fractions with Diaflo Membrane XM/100. Table III shows that the fractions of less than 100,000 mol. wt. (" antigen " fraction),

TABLE III.—*Absorption of Antibody in Hyperimmune Serum by " Antigen " Fraction Obtained by Dissociating Antibody–Antigen Complex in Sera of Tumour-bearing Animals*

| Sera from which Ag fraction had been obtained | No. of different sera tested by | | No. of fractions which absorbed antibody activity as measured by | |
|---|---|---|---|---|
| | MHI | FI | MHI | FI |
| Control | 3 | 9 | 0 | 0 |
| MCl tumour-bearing sera | 4 | 13 | 3 | 10 |
| MCl tumour-bearing sera normal pH | 2 | 4 | 0 | 0 |
| Rats bearing other tumours | 3 | 4 | 1 | 1 |
| MCl tumour-bearing irradiated | 3 | 4 | 1 | 1 |

whether obtained by treatment of the tumour-bearing serum with low pH or high molar salt solutions, were very effective in neutralizing the capacity of the tumour-immune serum to cause immunofluorescence or mixed cell haemadsorption. In contrast, in tumour-bearing serum not subjected to Ag–Ab splitting techniques, the separated low mol. wt. fraction did not lower the FI or MHI. Also, the high mol. wt. fraction did not show any antigenic activity, as tested by ability to inhibit the antibody in the tumour-immune serum. Table II and III indicate that for the MC–1 tumours at least, TSTA is released into the circulation and part of the antigenic activity is neutralized by a substance of mol. wt. greater than 100,000, from which it can be dissociated by lowering the pH or raising the molarity of the solution. These data are consistent with the hypothesis that tumour-bearing serum contains antigen–antibody complexes with antigen in excess.

*Inability to detect circulating TSTA in serum of immunosuppressed tumour-bearing rats*

Since the experiments described in the previous section have shown that part of the antigenic activity of the tumour-bearing serum is blocked by antibody, it seemed that one might raise the antigenic activity of the tumour-bearing serum by growing tumours in immunosuppressed animals. Consequently, rats were given 500 rad of total body x-irradiation followed by MC–1 tumour implantation. The sera from these animals after 10–12 days gave no evidence of circulating TSTA. Only at 21 days were any significant results obtained (Table II). Table III shows that even after the sera had been acidified and an " antigen " fraction separated, no indication of antigenic activity could be found. This unexpected finding indicates that for the MC–1 tumour, high levels of circulating TSTA are not reached with normal metabolic cell surface turnover and it would appear that a local immune reaction is necessary before measurable quantities of antigen are released from tumours into the circulation.

### DISCUSSION

The MC–1 chemically induced fibrosarcoma is highly antigenic and it is possible to produce considerable resistance of animals to this tumour by immunization. The tumour-specific antigen responsible for this graft resistance, the TSTA, has been shown to evoke the production of a specific antibody in the syngeneic animal, but the antibody activity in the serum of a tumour-bearing animal is masked by the release into the circulation of soluble TSTA which form antigen–antibody complexes. No evidence has been obtained to indicate that the serum of tumour-bearing animals is ever in antibody excess. Our series of experiments would indicate that the release of antigen in quantities measurable by our techniques requires an active immune reaction and this is clearly a phenomenon that has to be investigated in more detail.

In man, a colonic tumour-associated antigen (CEA), has been shown by radioimmunoassay techniques to be released into the circulation (Thomson *et al.*, 1969), and also soluble HL–A antigens have been demonstrated in human sera (Charlton and Zmijewski, 1970). Studies of the metabolism of the surface membrane molecules have shown a continuous cell surface turnover and release of substances into the surrounding medium (Warren and Glick, 1968). Our studies appear to indicate that the local immune reactions increase this cell surface turnover and subsequent release.

After excision of the tumour, circulating antigen is rapidly cleared and anti-TSTA antibody levels build up so that in the 7–14 days post-excision serum fairly high titres of specific antibody can be detected. The presence in the serum of tumour-bearing animals of both free antigen and antigen–antibody complexes may influence the host reaction directed against the tumour in a number of ways. The suggestion has already been made that in the presence of excess antigen the migration of immunoblasts from the draining node may be blocked (Hall and Morris, 1965). Immunoblasts are, however, only one component in the cell-mediated response of the host against the tumour; small memory-type lymphocytes and macrophages are also involved. It is possible that the circulating antigen and/or antigen–antibody complexes may, by directly interacting with the lymphoid cells or macrophages, interfere with their capacity to kill the specific target tumour cell. Vaage (1972) has shown that host resistance to tumour cell challenge can be depressed by giving killed tumour cells during the first week of tumour challenge. The depression of host resistance by antigenic tumour tissue appears to be the result of excess antigen and not " blocking " antibody. If this artificially induced antigen excess can depress *in vivo* rejection of a tumour challenge, the demonstration of the presence of excess circulating TSTA in the tumour-bearing animal may be interpreted as having a similar adverse effect. In view of our results, the recent report by Bansal and Sjögren (1971) of sera with " unblocking " activity in polyoma virus-induced tumours in rats is of interest. Here, sera from immune rats counteracted the blocking effect of sera from animals with progressively growing tumours on cell-mediated specific cytotoxicity. Moreover, such sera induced regression of 4 out of 5 transplanted tumours. The mechanism of the " unblocking " is not known but it was postulated that *in vitro* competition occurred between " blocking " and " unblocking " antibodies on the surface of the target tumour cell. An alternative hypothesis is that an excess of high affinity anti-TSTA antibody is achieved which clears the excess soluble antigen and reverses the " blocking " induced by this antigen–antibody complex in the tumour-bearing serum.

The results of the present investigation should also raise important questions of the efficiency of some of the present immunotherapy programmes in human cancer. Here, tumour cells may be injected in a situation where there is already tumour antigen excess, thereby increasing the antigen load and further suppressing the immune response. In cancer immunotherapy the removal of excess circulating antigen by administration of antibody might allow the host response to be maximally active in tumour rejection.

We wish to acknowledge the technical assistance of Miss Valerie Sellens. This work was supported by grants from the Medical Research Council and the Cancer Research Campaign. D.M.P.T. was in receipt of a Canadian Medical Research Council Fellowship and a Quebec Cancer Foundation Grant.

### REFERENCES

ALEXANDER, P. & HALL, J. G. (1969) The Role of Immunoblasts in Host Resistance and Immunotherapy of Primary Sarcomata. *Adv. Cancer Res.*, **13**, 1.

AVRAMEAS, S. & TERNYNCK, T. (1969) The Cross-linking of Proteins with Glutaraldehyde and its Use for the Preparation of Immunoabsorbents. *Immunochemistry*, **6**, 53.

BALDWIN, R. W. (1955) Immunity to Methylcholanthrene-induced Tumours in Inbred Rats following Atrophy and Regression of the Implanted Tumour. *Br. J. Cancer*, **9**, 652.

BALDWIN, R. W. & BARKER, C. R. (1967) Demonstration of Tumour-specific Humoral Antibody against Amino Azo Dye induced Rat Hepatoma. *Br. J. Cancer*, **21**, 793.

BANSAL, S. C. & SJÖGREN, H. O. (1971) " Unblocking " Serum Activity *in vitro* in the Polyoma System May Correlate with Anti-tumour Effects of Antiserum *in vivo*. *Nature, Lond.*, **233**, 76.

CHARLTON, R. K. & ZMIJEWSKI, C. M. (1970) Soluble HL–A Antigen: Localization in the $\rho$-Lipoprotein Fraction of Human Serum. *Science, N.Y.*, **170**, 636.

EVANS, R. & ALEXANDER, P. (1972) Role of Macrophage in Tumour Immunity. *Immunology*, **23**, 615, 627.

FISHER, G. A. (1958) Studies of the Culture of Leukaemic Cells *in vitro*. *Ann. N.Y. Acad. Sci.*, **76**, 673.

FOLEY, E. J. (1953) Antigenic Properties of Methylcholanthrene-induced Tumours in Mice of the Strain of Origin. *Cancer Res.*, **13**, 835.

HADDOW, A. & ALEXANDER, P. (1964) An Immunological Method of Increasing the Sensitivity of Primary Sarcomas to Local Irradiation with X-rays. *Lancet*, i, 452.

HALL, J. G. & MORRIS, B. (1965) The Immediate Effect of Antigens on the Cell Ouput of a Lymph Node. *Br. J. exp. Path.*, **46**, 450.

HARDER, F. H. & MCKHANN, C. F. (1968) Demonstration of Cellular Antigens in Sarcoma Cells by an Indirect $^{125}$I-labelled Antibody Technique. *J. natn. Cancer Inst.*, **40**, 231.

HELLSTRÖM, I. & HELLSTRÖM, K. F. (1970) Immunological Enhancement as Studied by Cell Culture Techniques. *A. Rev. Microbiol.*, **24**, 373.

MIKULSKA, Z. B., SMITH, C. & ALEXANDER, P. (1966) Evidence for an Immunological Reaction of the Host Directed Against its Own Actively Growing Primary Tumour. *J. natn. Cancer Inst.*, **36**, 29.

OLD, L. J. & BOYSE, E. A. (1964) Immunology of Experimental Tumours. *A. Rev. Med.*, **15**, 167.

OLSEN, R. G., MCCAMMON, J. R. & YOHN, D. S. (1970) Simplified Procedure for Preparation of Specific Antibodies to Gamma Globulins. *Appl. Microbiol.*, **20**, 75.

PILCH, Y. H. & RIGGINS, R. S. (1966) Antibodies to Spontaneous and Methylcholanthrene-induced Tumors in Inbred Mice. *Cancer Res.*, **26**, 871.

SJÖGREN, H. O., HELLSTRÖM, I., BANSAL, S. C. & HELLSTRÖM, K. E. (1971) Suggestive Evidence that the " Blocking " Antibodies of Tumour-bearing Individuals may be Antigen–Antibody Complexes. *Proc. natn. Acad. Sci.*, **68**, 1372.

SUNDQVIST, K. G. & FAGRAEUS, A. (1972) A Sensitive Isotope Modification of the Mixed Haemadsorption Test Applicable to the Study of Prozone Effects. *Immunology*, **22**, 371.

TACHIBANA, T., WORST, P. & KLEIN, E. (1970) Detection of Cell Surface Antigens on Monolayer Cells. *Immunology*, **19**, 809.

THOMSON, D. M. P., KRUPEY, J., FREEDMAN, S. O. & GOLD, P. (1969) The Radioimmunoassay of Circulating Carcinoembryonic Antigen of the Human Digestive System. *Proc. natn. Acad. Sci.*, **64**, 161.

VAAGE, J. (1972) Specific De-sensitization of Resistance Against a Syngeneic Methylcholanthrene-induced Sarcoma in C3HF Mice. *Cancer Res.*, **32**, 193.

WARREN, L. & GLICK, M. C. (1968) Membranes of Animal Cells. II. The Metabolism and Turnover of the Surface Membrane. *J. Cell. Biol.*, **37**, 729.